# Structure-Based Virtual Screening and In Vitro Evaluation of New *Trypanosoma cruzi* Cruzain Inhibitors

**DOI:** 10.3390/ijms20071742

**Published:** 2019-04-09

**Authors:** Verónica Herrera-Mayorga, Edgar E. Lara-Ramírez, Karla F. Chacón-Vargas, Charmina Aguirre-Alvarado, Lorena Rodríguez-Páez, Verónica Alcántara-Farfán, Joaquín Cordero-Martínez, Benjamín Nogueda-Torres, Francisco Reyes-Espinosa, Virgilio Bocanegra-García, Gildardo Rivera

**Affiliations:** 1 Laboratorio de Biotecnología Farmacéutica, Centro de Biotecnología Genómica, Instituto Politécnico Nacional, Reynosa 88710, Mexico; veronica_qfb@hotmail.com (V.H.-M.); frelibi@hotmail.com (F.R.-E.); vbocanegg@hotmail.com (V.B.-G.); 2 Departamento de Ingeniería Bioquímica, Unidad Académica Multidisciplinaria Mante, Universidad Autónoma de Tamaulipas, Mante 89840, Mexico; 3 Unidad de Investigación Biomédica de Zacatecas, Instituto Mexicano del Seguro Social (IMSS), Zacatecas 98000, Mexico; elarar0700@hotmail.com; 4 Departamento de Parasitología, Escuela Nacional de Ciencias Biológicas, Ciudad de México 11340, Mexico; perez_fabiola@outlook.com (K.F.C.-V.); bnogueda@hotmail.com (B.N.-T.); 5 Departamento de Bioquímica, Escuela Nacional de Ciencias Biológicas, Instituto Politécnico Nacional, Ciudad de México 11340, Mexico; charmina_burana@hotmail.com (C.A.-A.); lorena_rpaez@yahoo.com.mx (L.R.-P.); veroalf@yahoo.com (V.A.-F.); gahan81@hotmail.com (J.C.-M.); 6 Unidad de Investigación en Infectología e Inmunología, Centro Médico Nacional La Raza, Instituto Mexicano del Seguro Social (IMSS), Ciudad de México 029990, Mexico

**Keywords:** *Trypanosoma cruzi*, molecular docking, cruzain, Zinc^15^, inhibitors

## Abstract

Chagas disease (CD), or American trypanosomiasis, causes more than 10,000 deaths per year in the Americas. Current medical therapy for CD has low efficacy in the chronic phase of the disease and serious adverse effects; therefore, it is necessary to search for new pharmacological treatments. In this work, the ZINC^15^ database was filtered using the *N*-acylhydrazone moiety and a subsequent structure-based virtual screening was performed using the cruzain enzyme of *Trypanosoma cruzi* to predict new potential cruzain inhibitors. After a rational selection process, four compounds, Z2 (ZINC9873043), Z3 (ZINC9870651), Z5 (ZINC9715287), and Z6 (ZINC9861447), were chosen to evaluate their in vitro trypanocidal activity and enzyme inhibition. Compound Z5 showed the best trypanocidal activity against epimatigote (IC_50_ = 36.26 ± 9.9 μM) and trypomastigote (IC_50_ = 166.21 ± 14.5 μM and 185.1 ± 8.5 μM on NINOA and INC-5 strains, respectively) forms of *Trypanosoma cruzi*. In addition, Z5 showed a better inhibitory effect on *Trypanosoma cruzi* proteases than S1 (STK552090, 8-chloro-N-(3-morpholinopropyl)-5H-pyrimido[5,4-b]-indol-4-amine), a known cruzain inhibitor. This study encourages the use of computational tools for the rational search for trypanocidal drugs.

## 1. Introduction

According to the World Health Organization (WHO), the Americas are one of the primary regions with a high prevalence of Chagas disease (CD). CD, also known as American trypanosomiasis, affects approximately 8 million people worldwide with more than 10,000 deaths annually, mainly in endemic areas of Latin America [1,2,3]. The causal agent for CD is the parasite *Trypanosoma cruzi (T. cruzi)*, which is transmitted mainly by triatomine insects to mammals, including human beings [4,5,6].

Trypanocidal therapy currently involves the use of two drugs: nifurtimox (Nfx) and benznidazole (Bzn). However, these drugs have several limitations that include uncertainty about their efficacy, poor tolerance, and severe adverse effects [7,8,9]. The low effectiveness of both drugs in the chronic phase of the disease, as well as the lack of interest of pharmaceutical companies in developing new drugs, has generated the need for new chemical compounds against *T. cruzi*.

A pharmacological target widely studied in *T. cruzi* is cruzain (Cz), which belongs to the family of proteases or peptide hydrolases. Proteases play an important and indispensable role in parasitic organisms, allowing them to participate in key catabolic functions such as parasite immunoevasion, encystment, exanthema, and tissue cell invasion [10]. Moreover, cysteine proteases of parasites have immunogenic properties that make them suitable targets for vaccine developments or as biomarker candidates [10].

Cz is the main cysteine protease of *T. cruzi*, which is expressed through all stages of the parasite life cycle. It allows parasite survival within the host. It also participates in penetration of the parasite into the host cell, in the digestion of immunoglobulins, and other varied biological functions [11,12,13]. The search for new Cz enzyme inhibitors continues to be very important because of their success in blocking the parasite life cycle in animal models [11,12,13].

**Figure 1:**
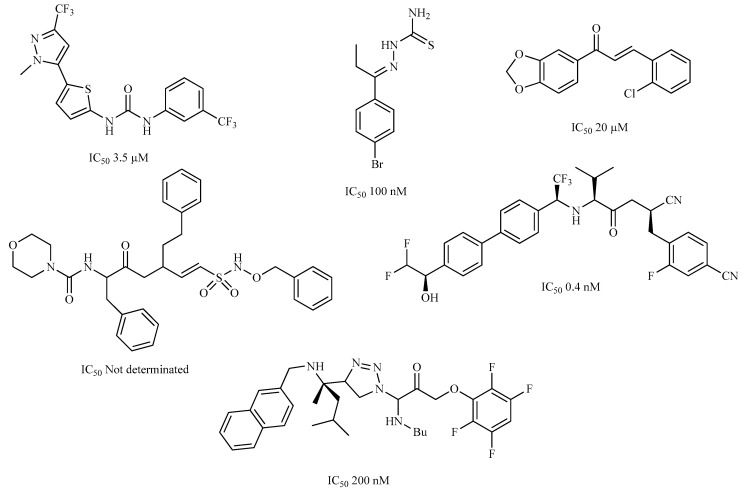
Chemical structure of urea, thiocarbazone, chalcone, amide, nitrile, and hydrazine derivatives identified as Cz inhibitors.

A variety of chemical structures identified as Cz inhibitors have been studied, such as those derived from ureas, carbazones, chalcones, carboxyamides, carboxylic acids, triazoles, vinylsulfones, hydrazones, and others (Figure 1) [14,15,16,17,18]. Among these compounds, thiazolylhydrazones have been proven powerful agents with a capacity to inhibit replication of the epimastigote (IC_50_ = 0.3 μM) with a higher trypanocidal activity than benznidazole (IC_50_ = 1.8 μM) [4]. Other authors state that hybrids of *N*-acylhydrazone and furoxane are active in amastigotes of *T. cruzi*. Additionally, this class of compounds, by permeability screening in Caco-2 cells and cytotoxicity assays in human cells, showed less cytotoxicity, good permeability, and a higher selectivity index than the reference drug benznidazole [19]. Additionally, the *N*-acylhydrazone moiety is considered a privileged structure in medicinal chemistry, since it has the potential to interact with different biological targets, including the *T. cruzi* cysteine protease [20,21].

In this work, the *N*-acylhydrazone moiety was used as the main filter in the ZINC^15^ database. This filtering step allowed obtention of a subset of chemicals in order to predict new potential Cz inhibitors by a structure-based virtual screening. Finally, four chemical compounds were chosen to test their trypanocidal activity in an in vitro model against epimastigotes and trypomastigotes of *T. cruzi* and their enzymatic inhibition effects in an extract of cysteine proteases.

## 2. Results and Discussion

### 2.1. Virtual Screening

After screening using the moiety (C=NNC(C)=O), a total of 2221 compounds that met our inclusion criteria were retrieved from the ZINC^15^ database. These compounds were cyclic *N*-acylhydrazone derivatives (Table S1, Table 1). The reason for this high number was because the small size of the moiety resulted in a high probability of it appearing during screening. However, we decided to continue the work with these compounds because they belong to the primary category known to be used as initial starting points in medicinal chemistry efforts [22]. This category is also one of the broadest libraries used in virtual screening analyses since these structures have simple chemical characteristics that are susceptible to chemical optimization [23]. Hence, these compounds were used in the next analysis.

**Figure 2:**
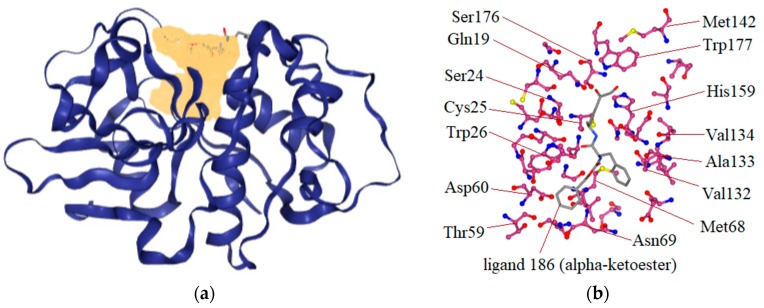
Binding site detection of the binding site on the crystallographic structure 1U9Q. This pocket, yellow region (**a**) was detected by DoGSiteScorer tool using ProteinsPlus server. (**b**) Interactions recorded in cruzain-ligand 186 complex.

Cz is a cysteine protease responsible for protein degradation. Its enzyme activity is related to the presence of cysteine (Cys25) and histidine (His172) residues in the active site. The kind of covalent bond formed between the ligand and active site at Cys25 is very important to determine if the inhibition is reversible or irreversible. For example, vinylsulfones and chalcones are covalently bound to the active thiol from Cys25 and act as irreversible inhibitors. In molecular docking analyses, the Cz structure 1U9Q with the ligand 186 (Table 1), a benzyl ester, was used. This was because in other studies, relevant interactions have been shown with amino acids of the catalytic triad (Cys25, His159, and Asn175) and with the amino acids Gln19, Asp158, Trp177, and Gly66, which have been reported in interactions showing a large series of hydrazone derivatives and competitive inhibitors of Cz [4,19,24]. With the ProteinsPlus server and the DoGSiteScorer tool, the binding site of 1U9Q was identified (Figure 2a). This site has a surface of 820.85 Aº, where 15 hydrogen donors, 37 hydrogen acceptors, 35 hydrophobic interactions, 47% non-polar amino acids, 33% polar amino acids, and 7% positively charged and 13% negatively charged amino acids are located. Residues contained in the pocket of the binding site are shown in Figure 2b.

The coordinates of this binding site in 1U9Q were used to dock the retrieved 2221 ligands. A total of 682 ligands showed a negative binding energy (<−6.6 Kcal/mol) lower than that obtained for ligand 186 (Table S1). Table 1 shows the best compounds (top ten with binding energies <−8.0 Kcal/mol) containing the moiety (C=NNC(C)=O).

**Figure 3:**
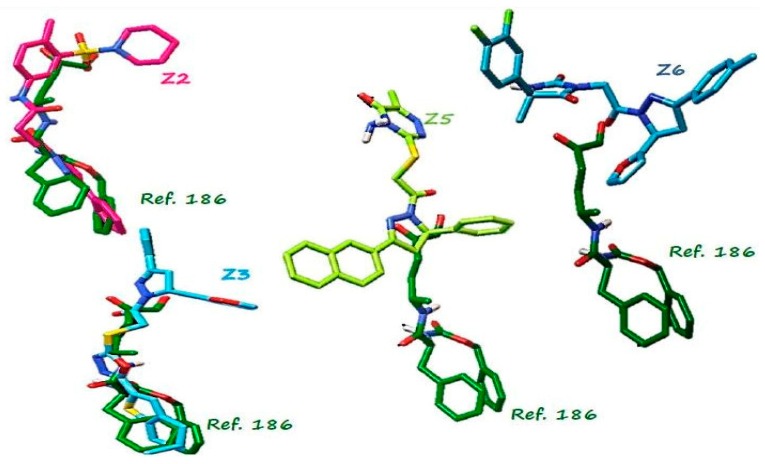
Analysis of coupling postures between the pose of ligand 186 interacting on the catalytic site (green) and compound Z2 in pink, Z3 in light blue, Z5 in green lemon and Z6 in blue, alignment of structures performed with AutoDock Tools programm.

The grouping analysis of 2221 ligands made with AuPosSOM showed 20 groups (Figure S1). No grouping pattern correlated with the binding energy, a finding similar to that reported by Fereidoonnezhad et al. [25]. In the present work, we selected compounds with the highest binding energy within each clade and reviewed their commercial availability, as previously done in [26]. Of the 20 clades (Figure S1), 9 contained compounds with the best scores distributed in groups 4, 6, 10, 11, 14, 16, 17, 18, and 19, and four groups contained compounds with lower scores (2, 12, 13, and 20). Within those 20 clades, 6 compounds Z1, Z2, Z3, Z4, Z5, and Z6 showed the highest negative scores (Table 1); however, the best compound, Z1, was not commercially available, therefore, it was not considered in further selection analyses. Compound Z4 was not considered for in vitro analyses either because it had a binding energy similar to Z3, and the interactions presented were very similar. A detailed visual inspection for amino acid interactions (Figure 3) was performed for compounds Z2 and Z3. We observed that both compounds interacted in the catalytic site in a manner similar to the reference inhibitor 186. The compounds Z5 and Z6 interacted in an opposite binding site, which indicated that they could be non-competitive inhibitors and warrant further Cz binding site explorations.

**Figure 4:**
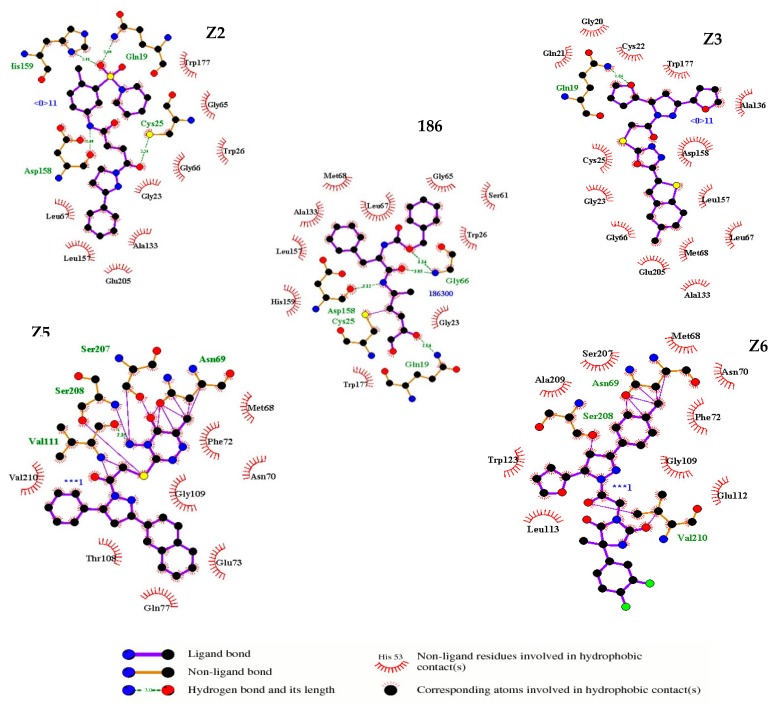
Amino acid interactions of the four (Z2, Z3, Z5 and Z6) selected compounds and the reference ligand 186 on Cz. In the five figures, arcs with red lines represent hydrophobic amino acid contacts, green dashed lines represent hydrogen bonds and purple lines represent covalent bonds between protein and ligand or “elastic” bonds within the ligand. The image was produced with LigPlot software.

The amino acid interactions for Z2 involved hydrophilic interactions with the amino acids His159 and Cys25; both amino acids are essential for the catalytic process. Other hydrophilic interactions observed for the Z2 compound near the catalytic site were Gly66, Gln19, and Asp158. In contrast, the compound Z3 only presented a hydrophobic interaction with the catalytic residue Cys25 as well as hydrophobic interactions with Gly66, Gln19, and Asp158. Both compounds Z2 and Z3 presented interactions through hydrophobic bonds with amino acids (Ala133, Leu157, Leu67, Gly23, Trp177, Gly66, and Glu205). On the other hand, the compound Z5 showed hydrophilic interactions with the residues Asn69, Ser208, Ser207, and Val111 as well as hydrophobic interactions with the residues Phe72, Asn70, Gly109, Glu73, Gln77, Trp108, and Val210. The compound Z6 showed hydrophilic interactions with Asn69, Ser208, and Val210 as well as hydrophobic interactions with Asn70, Phe72, Gly109, Glu112, Leu 113, Trp123, Ala209, and Ser207. Most of the hydrophilic interactions with the amino acids Cys25, His159, Gln19, Ser207, and Val210 are involved in the binding of oxygen atoms with the carbonyl groups present in the four compounds; this class of interactions has been reported for irreversible Cz inhibitors that contain ketones. Such chemical reactions provide the electrophilia necessary to interact with the enzyme conferring the mechanism of enzyme inhibition to elapse the reaction [27]. Moreover, it was also observed that the residues Asn69, Ser208, and Asp158 interacted with hydrogen atoms present in the methyl group and NH groups because they were polar acceptor residues or proton donors. Compounds Z5 and Z6 presented interactions with residues Asn69, Ser208, Ser207, Val111, and Val210, which are represented as covalent bonds (Figure 4). Based on the above findings, the compounds Z2, Z3, Z5, and Z6 were selected for subsequent experimental studies.

### 2.2. In Vitro Evaluation

The compounds S1 (STK552090, 8-chloro-N-(3-morpholinopropyl)-5H-pyrimido[5,4-b]-indol-4-amine, a known Cz inhibitor), Z2, Z3, Z5 and Z6 were evaluated in vitro as potential trypanocidal agents against epimastigotes (INC-5 strain) and trypomastigotes (NINOA and INC-5 strains) of *T. cruzi*, using Bzn and Nfx as reference drugs. The results are shown in Table 2. Bzn had a better trypanocidal effect (LC_50_ = 9.64 µM) than Nfx (LC_50_ = 38.36 µM) in the epimastigote. In the trypomastigote, however, the best biological activity was produced by Nfx (LC_50_ < 117.16 µM) in both strains. This was an expected variance for the reference drugs studied in vitro and in vivo [28,29,30,31]. The variation between different parasitic stages could be because the epimastigote is not the infectious stage. Its susceptibility is not reflected in the other stages (trypomastigotes and amastigotes) or to a behavior clearly dependent on the dose, in both parasitic stages, to cell viability [29]. In other words, therapeutic success seems to depend on the susceptibility of the parasites to the trypanocidal drug, the access and accumulation of the drug in different environments (plasma levels and specific tissues), and the host immune response [30].

The evaluated compounds Z2, Z3, Z5, and Z6 showed different trypanocidal effects. In the epimastigote form, the compounds Z3 and Z6 did not show trypanocidal effects (LC_50_ > 250 µM). The compound S1, a Cz inhibitor, also did not show an effect. Compounds Z2 and Z5 only showed trypanocidal activity. Z2 had a low activity, but Z5 showed a trypanocidal effect similar to Nfx, though it was four times less potent than Bzn.

Biological behavior did not vary between the compounds described above and the form of *T. cruzi* evaluated. Against bloodstream trypomastigotes, the compounds Z2, Z3, Z6 and the inhibitor S1 showed LC_50_ values greater than 250 μM, resulting in a concentration twice as high as that obtained with the reference drugs for both strains (INC-5 and NINOA). The trypanocidal results obtained from the S1 inhibitor differ from that reported by Ferreira et al. (2010) and Pinto et al. (2017), because these authors indicated that the compound S1 showed IC_50_ values of 2.5 μM in infected mouse fibroblasts (L929) with trypomastigotes of *T. cruzi* of the Tulahúen strain.

The most active compound in both stages and both strains was compound Z5, which in epimastigotes of the strain INC-5, showed the same trypanocidal activity as that of Nfx. On the other hand, in trypomastigotes, Z5 presented LC_50_ values lower than Bnz in both strains with concentrations 1.6 times lower than those present with the drug Nfx; therefore, compound Z5 is a promising structure in the search for new agents to treat Chagas disease.

### 2.3. Enzyme Inhibition

To confirm the predictive study of new potential Cz inhibitors and confirm the mechanism of action, enzymatic inhibition with cysteine proteases of *T. cruzi* was done. The results of the mean inhibitory concentration of the enzyme activity are shown in Table 3. A behavior similar to that of the in vitro evaluation on epimastigotes and trypomastigotes of *T. cruzi* can be seen. The compounds Z2 and S1 showed weak inhibitory activities (IC_50_ > 200 µM). Z3 showed an IC_50_ value of 84.37 µM in protease inhibition, but this compound did not have a trypanocidal effect in epimastigotes and trypomastigotes. In this evaluation, we observed that the compounds Z5 and Z6 were characterized by a better inhibitory activity with IC_50_ values of 56.23 μM and 50.35 μM, respectively. However, Z6 also did not have a trypanocidal effect. In contrast, Z5 was the best compound with trypanocidal activity against epimastigotes and trypomastigotes. Although these results confirm an inhibition of cysteine proteases as mechanism of action, a specific study on the Cz enzyme is necessary to determine the kind of inhibition that these compounds could have.

**Figure 5:**
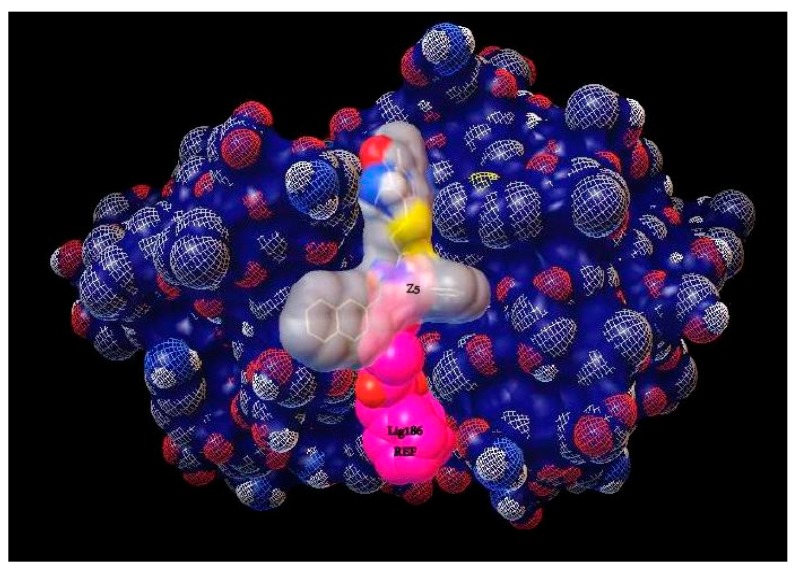
Analysis of the interaction site between ligand 186 and compound Z5 on Cz.

According to the results obtained in this work, Z5 presents interesting biological activity that provides a guide to analyze analogous compounds as well as to continue analyzing the interactions and the place where these occur since, as can be seen in Figure 5, it is a different catalytic site. This could possibly favor the activity of compound Z5.

For the above, the binding mode and enzyme activity were not correlated, as we expected. The lack of biological relation could be due to the physicochemical properties of the molecules. This could interfere in the penetration of compounds into cell membranes or limit evaluation of Cz enzyme inhibition on *T. cruzi* only, not in an extract of proteases as we did [11].

## 3. Materials and Methods

### 3.1. Structure-Based Virtual Screening

Compounds were selected from the clean lead folder (*n* = 4,591,276 ligands) available in the ZINC^15^ database (http://zinc.docking.org, accessed on: 5 August 2018). Filtration of the clean lead compounds was done using the general structure of an *N*-acylhydrazone, with the format formula (C=NNC(C)=O), including chemical structures that had up to 50% similarity and complied with Lipinski’s rules. Ligands that met the criteria were downloaded and prepared as ligands by adding hydrogens and a subsequent energy minimization process with the obminimize command from Babel software [32]. Then, Gasteiger charges were added during the transformation to pdbqt files with the “prepare_ligand4.py” script of AutoDock Tools [33]. The ligands in pdbqt format were used for the further structure-based virtual screening through a molecular docking analysis with the software Autodock vina. For molecular docking, the three-dimensional structure of Cz (PDBID: 1U9Q) obtained from the Protein Data Bank database (http://www.pdb.org, accessed on: 15 July 2018) was used. This structure was used as a receptor to remove the ligand 186 (1-(1-methyl-4,5-dioxo-pent-2-enylcarbamoyl)-2-phenyl-ethyl]-carbamic acid benzyl ester) and the water molecules. Then, the receptor molecule was prepared using the AutoDock Tools software, also by the addition of polar hydrogens, Gasteiger charges, and the generation of the receptor in pdbqt format.

To determine the size of the search spaces in the active site of Cz, the online program Proteins Plus and the tool DoGSiteScorer https://proteins.plus/ (accessed on 11 December 2018) were employed to confirm the binding site in order to design the most optimal grid-box [34]. With the obtained information, a grid-box was determined with the following sizes: X = 18 Å, Y = 15 Å, and Z = 20 Å and with the center coordinates X = 7.605, Y = 9.788, and Z = 8.779. This search space was used for docking the retrieved ZINC^15^ compounds. The binding energy score obtained from the first reference docking with ligand 186 was used as a cutoff value for the selection of potential inhibitors on the basis of the most negative energy binding higher than that of ligand 186. A total of 682 ligands were chosen because these showed the best binding energies (<−6.6 Kcal/mol). The compounds were further analyzed according to their amino acid contact with Cz, using the software AuPosSOM (Automatic analysis of poses using SOM) [35], with the aim of generating clusters that could be related across the binding energy and the structure of the ligand. The AuPosSOM results contained in the Newick tree file were plotted with TreeGraph2 [36] and analyzed visually. In addition, a more detailed analysis of the interactions between the best selected compounds that docked onto the protein was performed using the LigPlot program [37].

### 3.2. In Vitro Evaluation

For biological evaluation, the compounds Z2 (ZINC9873043), Z3 (ZINC9870651), Z5 (ZINC9715287), and Z6 (ZINC9861447) were selected and purchased from Molport https://www.molport.com (accessed on: 03 March 2018). For evaluation of trypanocidal activity of the selected compounds, two strains (NINOA and INC-5) in the epimastigote or trypomastigote stage of *T. cruzi* were used. Each strain was used to infect CD-1 mice (18–20 g) intraperitoneally with a concentration of 1 × 10^6^ trypomastigotes/mL of blood. In the maximum peak of parasitemia, blood was obtained by cardiac puncture using heparin as an anticoagulant. The parasite concentration was then adjusted with isotonic saline (0.85% NaCl), and 90 μL of blood was used to achieve a concentration of 1 × 10^6^ trypomastigotes/mL of blood. A 10 μL sample of each compound was evaluated in 96-well plates. The treatments performed consisted of a negative control, which contained dimethylsulfoxide (DMSO 2.5%) and the four compounds selected from virtual screening. As positive controls, the reference drugs Bnz (Rochagan, Roche) and Nfx (Lampit, Bayer) were used as well as the compound STK552090 (8-chloro-N-(3-morpholinopropyl)-5H-pyrimido[5,4-b]indol-4-amine), which was named internally as S1 and reported as a Cz inhibitor in *T. cruzi* in the ZINC^15^ library. Once the selected compounds were added after being homogenized with blood, the plates were incubated at 4 °C for 24 h [38]. Once the incubation time had elapsed, the plates were kept at room temperature for 30 min, and then an aliquot of 5 μL was taken from each well, a fresh preparation was made, and the viable trypomastigotes were counted using the Pizzi method [39,40]. A calculation of the number of viable trypomastigotes per mL of blood was carried out, and the survival percentages were obtained taking 100% of the negative control. Each test was done in triplicate. To obtain 50% lysis concentration (LC_50_) of the population, the following concentrations were used: 200, 100, 50, 25, and 12.5 μg/mL. The LC_50_ values were determined using Probit statistical analysis of the dose-response, and the results were expressed as the mean of the standard deviation. The results were later converted to micromolar units.

### 3.3. Enzyme Inhibition

*T. cruzi* epimastigotes of the INC-5 strain were used to obtain a protein extract quantified by the Bradford method. Evaluation of the activity was carried out using the synthetic fluorogenic peptide Z-Phe-Arg-MCA as a substrate at 5 µM, with 1 μg of the enzyme extract and the corresponding compounds (Z2, Z3, Z5, and Z6) at different concentrations (6.25–200 µM) using a reaction buffer (50 mM Na_2_HPO_4_, 100 mM NaCl, 5 mM EDTA, 2.5 mM dithiothreitol; pH 6.5) at room temperature. As a negative control of enzyme inhibition, the substrate without compounds was evaluated with 2% DMSO (concentration corresponding to the highest dilution of the compounds). Hydrolysis of the substrate was monitored in a continuous assay for 1 h in the Spectramax M5 spectrofluorometer (Molecular Devices) at 380 nm excitation and 440 nm emission. The mean of fluorescence changes was normalized taking the control (without inhibitor) as 100%. The IC_50_ values were determined from semilog concentration-response plots using the nonlinear curve-fitting method [41].

The relationship of the biological evaluation with the binding mode of the selected molecules was inferred based on in vitro results.

## 4. Conclusions

Filtering the ZINC^15^ database using the N-acyl hydrazone moiety and further structure-based virtual screening allowed us to select four compounds as potential Cz enzyme inhibitors with trypanocidal activity. Compounds Z5 showed the best trypanocidal activity, with LC_50_ values in epimastigotes of 36.26 ± 9.9 μM and in trypomastigotes of 166.21 ± 14.5 μM for the strain NINOA, and 185.1 ± 8.5 μM for the strain INC-5. In addition, this compound showed inhibition of *T. cruzi* proteases with an IC_50_ value of 56.23 μM. According to the in silico study of its interaction in the active site of the enzyme, Z5 could be a non-competitive inhibitor. Therefore, the results obtained encourage us to continue using computational tools to search for anti-*Trypanosoma cruzi* drugs and to continue exploring the behavior of these derivatives on the Cz enzyme.

## Figures and Tables

**Table 1:** Structures of the top ten compounds with the best binding energies obtained from a clean lead database of ZINC^15^.

Compound	Structure	Binding Energy (kcal/mol)	Activity Reported
Z1 ZINC129684040	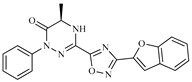	−8.6	None
Z2 ZINC9873043	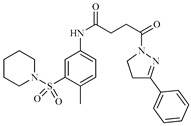	−8.3	Inhibitor of cannabinoid receptor type 2
Z3 ZINC9870651	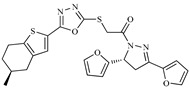	−8.2	None
Z4 ZINC9835465	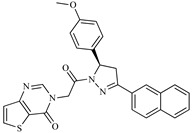	−8.2	Amine oxidase inhibitor
Z5 ZINC9715287	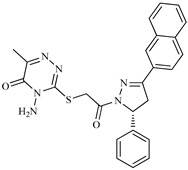	−8.1	Amine oxidase inhibitor
Z6 ZINC9861447	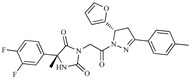	−8.1	Amine oxidase inhibitor
Z7 ZINC9873693	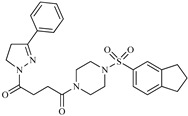	−8.1	Inhibitor of aldo keto reductases
Z8 ZINC14741665	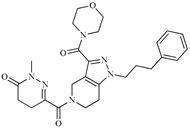	−8.0	Inhibitor of cathepsin S
Z9 ZINC60293658	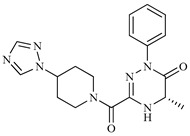	−8.0	None
Z10 ZINC9867137	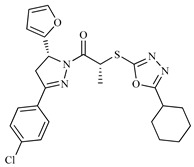	−8.0	Amine oxidase inhibitor
186	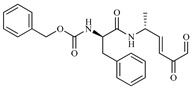	−6.6	Cz inhibitor

**Table 2:** Half maximal lytic concentration of the compounds Z2, Z3, Z5, Z6 and S1 and the reference drugs on epimastigotes and trypomastigotes of *T. cruzi*.

Compound	Epimastigotes	Bloodstream Trypomastigotes	
	LC_50_ (µM) *T. cruzi* INC-5	LC_50_ (µM)	
		*T. cruzi* NINOA	*T. cruzi* INC-5
Z2	239.40 ± 9.3	>250	>250
Z3	>250	>250	>250
Z5	36.26 ± 9.9	166.21 ± 14.5	185.1 ± 8.5
Z6	>250	>250	>250
S1	>250	>250	>250
Nfx	38.36 ± 5.2	99.41± 11.1	117.16 ± 16.36
Bnz	9.64 ± 4.2	183.1 ± 16.2	225.40 ± 26.5

**Table 3:** IC_50_ values for cysteine proteases from epimastigotes of *Trypanosoma cruzi* strain INC-5.

Compound	IC_50_ (µM)
Z2	1410.05
Z3	84.37
Z5	56.23
Z6	50.35
SI	>200

## Supplementary Materials

Supplementary materials can be found at https://www.mdpi.com/1422-0067/20/7/1742/s1. Figure S1: Clustering pattern obtained with AuPosSOM based on the amino acid-ligand contact footprint. Table S1: Compounds obtained from virtual screening, binding energies and chemical properties.

## Abbreviations

Nfx	Nifurtimox
Bzn	Benznidazole
Cz	Cruzain
CD	Chagas disease
IC_50_	Inhibitory concentration
LC_50_	Lytic concentration
