# Supplementary material for: Structure-Based Virtual Screening and In Vitro Evaluation of New Trypanosoma cruzi Cruzain Inhibitors
Source: Int J Mol Sci. 2019 Apr 9;20(7):1742. doi: 10.3390/ijms20071742 (PMC6479639; doi:10.3390/ijms20071742)

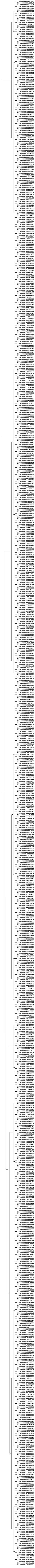

Supplement: Supplementary file 1 [file Figura_S1_tree.pdf › Nueva carpeta/Figura_S1_tree.pdf]
